# Hemodynamics of the oral mucosa during cooling: A crossover clinical trial

**DOI:** 10.1016/j.heliyon.2023.e19958

**Published:** 2023-09-07

**Authors:** J. Walladbegi, J.E. Raber-Durlacher, M. Jontell, D.M.J. Milstein

**Affiliations:** a Department of Oral Medicine and Pathology, Institute of Odontology, The Sahlgrenska Academy, University of Gothenburg, Gothenburg, Sweden; b Department of Oral and Maxillofacial Surgery, Amsterdam University Medical Center, Amsterdam, the Netherlands; c Department of Oral Medicine, Academic Center for Dentistry Amsterdam (ACTA), Amsterdam, the Netherlands

**Keywords:** CytoCam, Intraoral cooling device, Microcirculation, Oral cryotherapy, Tissue oxygen saturation

## Abstract

**Objective:**

Oral cryotherapy is used to prevent the onset of oral mucositis, a common and debilitating adverse effect following cancer chemotherapy. A protective mechanism associated with oral cooling is thought to be mediated through reduced tissue microcirculation. The aim of the present study was to examine the underlying mechanism associated with oral mucosal cooling by measuring oral microcirculation and tissue oxygen saturation after cooling with ice chips (IC) and an intraoral cooling device (ICD).

**Study design:**

In a single-center randomized crossover study, 10 healthy volunteers were assigned (1:1) randomly to the order in which the two intraoral cooling procedures (IC/ICD) were to be commenced. On day 1, half of the study participants started with IC and then crossed over to intraoral cooling with the ICD on day 2, while the other half of the participants undertook the same two procedures in the reverse order. Total and functional capillary density (T/FCD) and tissue oxygen saturation (StO_2_) measurements were obtained at baseline and 30 min following oral cooling.

**Results:**

Following 30 min of oral cooling, a statistically significant difference was found for FCD between IC and ICD (percentage points; +2 vs. −13; *p* < 0.05). A statistically significant decrease in StO_2_ was observed with both IC and ICD (%; 13 vs. 10) after 30 min of cooling as compared to baseline (*p* < 0.05). As for the participants’ preference the ICD was preferred over IC by 9 out of 10 participants (*p* = 0.021).

**Conclusions:**

Both microcirculation parameters and tissue oxygen saturation are altered in conjunction with oral cooling, indicating their potential mechanistic contribution towards cryoprevention of oral mucositis.

## Highlights

• Real-time visualization of the oral microcirculation using incident darkfield imaging (CytoCam Video Microscope System).
• Microcirculatory parameters and tissue oxygen saturation are altered in conjunction with oral cooling.
• Mapping the mucosal events during oral cooling could be a valuable footing for the development of new treatment strategies for prevention of oral mucositis.

## Abbreviations

5-FU: 5-fluorourcil
BMI: body mass index
CC: CytoCam
CyT(s): cryotherapy(-ies)
DBP: diastolic blood pressure
FCD: functional capillary density
Fd: focal depth
HR: heart rate
IC: ice chips
ICD: intraoral cooling device
ISTOM: InSpectra™ StO_2_ Tissue Oxygen Monitor
MAP: mean arterial pressure
MFI: microvascular flow index
NIRS: near-infrared spectroscopy
OCyT(s): oral cryotherapy(-ies)
OM: oral mucositis
PP: percentage points
RBM: right buccal mucosa
RLLM: right lower lip mucosa
ROI(s): region(s) of interest
RULM: right upper lip mucosa
SBP: systolic blood pressure
SpO_2_: peripheral capillary oxygen saturation
StO_2_: tissue oxygen saturation
TCD: total capillary density
THI: tissue hemoglobin index

## 1 Introduction

At present the mechanisms by which oral cryotherapy (OCyT) preserves the oral mucous membrane when exposed to chemotherapeutic drugs are not fully elucidated. Vasoconstriction of the areas subjected to cooling is the most accepted model, which leads to reduced blood flow and tissue exposure to chemotherapeutic cytotoxic agents [1]. Cryotherapy (CyT) is used within various medical disciplines to manage and limit the impact of pathophysiology concerning life threating conditions such as major stroke, severe traumatic brain injury and cardiac arrest [2]. CyT is used within the field of hematology and oncology to reduce the duration and severity of chemotherapy-induced oral mucositis (OM), a common and debilitating adverse effect following chemotherapy [[3], [4], [5]]. In its mildest form OM is characterized by painful erythema and can worsen and result in diffuse ulcerations. The compromised oral mucosa usually heals within two weeks if it does not succumb to a secondary infection [6], which may then lead to sepsis and in its most severe outcome, fatality [[7], [8], [9], [10]]. Based on a comprehensive review of the literature, the Multinational Association of Supportive Care in Cancer/International Society of Oral Oncology (MASCC/ISOO) recommends OCyT as a strategy for prevention of OM in patients receiving 5-fluorourcil (5-FU) or high-dose melphalan as conditioning regimens for hematopoietic stem cell transplantation (HSCT) [11].

OCyT using ice chips (IC) is the most extensively investigated strategy for prevention of OM and has significantly demonstrated efficacy in previous studies [12,13]. Nevertheless, despite these positive observations IC can result in unpleasant experiences including chills, headache, numbness/taste disturbance, teeth sensations and nausea [1,14,15]. To counteract the potential discomforts of cooling with IC an intraoral cooling device (ICD) was developed. The ICD proved to be equally effective as IC in terms of oral temperature reduction but was significantly better tolerated and accepted compared to IC.16 Furthermore, conventional cooling with IC refined and further improved using an ICD in prevention of OM in lymphoma patients [16].

To better understand the underlying mechanism by which OCyT protects the oral mucosa, oral microcirculation and tissue oxygen saturation measurements were compared between IC and the ICD. It is hypothesized that the installation of an ICD yields better overall oral cooling by reducing mucosal capillary density compared to swirling IC intraorally. Thus, the aim of the present study was to examine the underlying mechanism associated with oral mucosal cooling by measuring oral microcirculation and tissue oxygen saturation after cooling with IC and the ICD.

## 2 Materials and methods

All the procedures in this study involving human participants were performed in accordance with the ethical principles established in the World Medical Association (WMA) Declaration of Helsinki (Fortaleza, October 2013). The study design and guidelines were further reviewed and approved by the Swedish Ethical Review Authority (Reference No. 2022-03386-01). All participants received information about the study procedures and signed informed consent was obtained.

### 2.1 Study volunteers

Inclusion was arbitrary and based on the availability of the participants. Subjects were considered eligible to participate in the study if they met the following inclusion criteria: willing and able to provide written informed consent, male or female, age ≥18 years, American Society of Anesthesiologist (ASA) classification 1, i.e., a normal healthy patient, did not use any types of medications that could impact the cardiovascular system, had no mucosal lesions or other oral pathologies that could affect the outcomes of the study. Exclusion criteria were: any previous history of head and neck malignancy or other intraoral pathologies, no previous history of radiation therapy to the head and neck, no use of tobacco or Swedish snuff.

### 2.2 Experimental design

This pilot study was a single-center randomized crossover investigation in healthy volunteers. Randomization was performed with a 1:1 allocation to assign the subjects randomly to the order in which two intraoral cooling procedures were to be commenced. On day 1, half of the study participants started with ice chips (IC) and then crossed over to the intraoral cooling device (ICD) on day 2, while the other half of the participants undertook the same two procedures in the reverse order. All cooling procedures continued for 30 min, i.e., 30 min cooling with ICs or ICD. A total of 4 measurement time points were obtained (day 1: baseline and after 30 min of cooling, day 2: baseline and after 30 min of cooling).

### 2.3 Basic hemodynamics

Mindray VS-600 Vital Signs Monitor (Shenzhen Mindray Bio-Medical Electronics Co., Ltd., Nanshan, Shenzhen, China) is a portable vital sign monitor for periodic spot-check and features simple operations to record basic systemic hemodynamics such as heart rate (HR; bpm), systolic blood pressure (SBP; mmHg), mean arterial pressure (MAP; mmHg), diastolic blood pressure (DBP; mmHg), peripheral capillary oxygen saturation (SpO_2_; %).

Genius™ 2 Tympanic Thermometer and Base (Covidien LLC, Mansfield, MA, USA) is an accurate ear canal thermometer with disposable single use probe covers and fast temperature acquisition. The range of measured temperature acquisitions is between 33° and 42 °C and are displayed with audible and visual indication on an easy-to-read liquid crystal display with icons.

### 2.4 Oral cryotherapy

Ice chips (IC) were produced in a commercial ice maker (Porkka KF145 Flake Ice Machine, Oulu, Finland) designed for hospital environment and stored in plastic containers at room temperature during the cooling procedures. IC temperature was approximately −0.5 °C upon exposure.

**Fig. 1:**
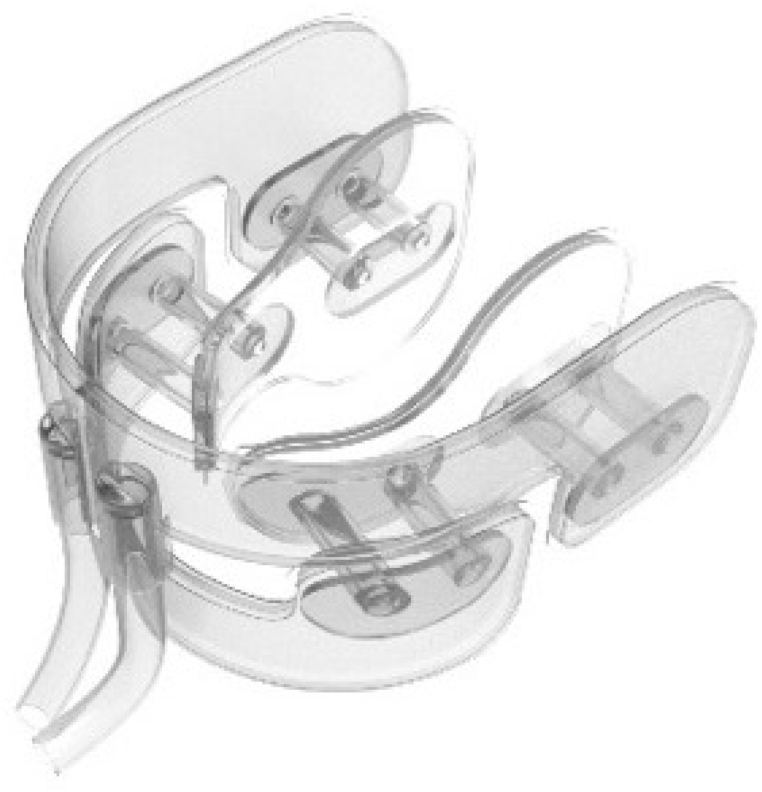
Schematic illustration of the intraoral cooling device.

The ICD (Cooral®Mouth device; Fig. 1) was provided by a Swedish medical technology company (BrainCool AB, Lund, Sweden) in two different sizes (small and medium). The ICD is a non-sterile single patient use device composed of biocompatible material (polyolefin polymer compound based on ethylene-vinyl acetate copolymer) that contain no latex or substances classified as hazardous. The ICD operates through a closed conduit system with continuously circulating cooled water. It is designed with compartments in order to provide cooling for the buccal mucosa, lips, floor of the mouth, tongue, gingiva and the hard palate. The cooled water was delivered via a portable thermostat unit (Cooral® System) that was connected to the ICD. The thermostat unit consisted of an energy control element with a computerized control system. Temperature-controlled cooled water was circulated from the unit through the conduits of the ICD with a flow rate between 0.25 and 0.28 L/min (±0.1 L/min), which resulted in cool temperature exchange between the cooling medium and the subjects’ oral mucosae. A mean temperature of 8° ± 2 °C was maintained during the entire cooling period and any deviations from the default temperature were automatically adjusted by the system [17,18].

### 2.5 Microcirculation imaging

Microcirculation measurements were performed using incident darkfield imaging (IDFI) (CytoCam Video Microscope System, Braedius Medical BV, Huizen, The Netherlands). Briefly, the CytoCam (CC) system comprises a lightweight, handheld instrument that operates by epi-illuminating the tissue of interest with pulsed (2 ms) green light at a wavelength of 530 nm. Since hemoglobin in erythrocytes absorb the green light, the remaining light scatters into the surrounding tissue and a high-resolution image (14 megapixel, 25 frames per second [fps]) of dark circulating globules circulating in blood vessel lumen is generated. These dark globules (red blood cells) in the lumen of blood vessels are contrasted by a bright background in a field-of-view equal to 1.80 mm^2^. The CC imaging probe is covered with disposable plastic cap (CytoCam Protection Caps, Braedius Medical BV, Huizen, The Netherlands) and is connected to a fanless medical grade panel personal computer (Braedius Medical BV, Huizen, The Netherlands) equipped with the CC Tools software (CytoCamTools Camera Manager v1.7.12, Braedius Medical BV, Huizen, The Netherlands) for camera operation and video data processing.

### 2.6 Microcirculatory data analysis

Capillary loop enumeration was performed using the Adobe Photoshop counting tool (Adobe Photoshop CC 2015, Adobe Systems Inc., San Jose, CA, USA). Prior to initiating capillary density measurements, the gray-levels of the images were standardized by using the levels tool available in Adobe Photoshop. Capillary density was analyzed by selecting a still frame from each recorded clip from each region of interest (ROI) according to established consensus criteria based on image resolution, brightness, clarity, and avoidance of microcirculation pressure artefacts [19,20]. Total capillary density (TCD) and functional capillary density (FCD) were assessed by counting the total number of capillaries and the number of functional capillary loops, i.e., capillaries with moving erythrocytes per visual field in each frame (area of 1.80 mm^2^). After completing capillary enumeration for each density data set, the results were divided by 1.80 to standardize and presented datasets as the mean number of capillaries per millimeter squared (cpll/mm^2^). In addition, to adjust for inherent biological variation between participants TCD and FCD were normalized with respect to baseline measurements and converted to percentages (%). Proportion of perfused capillaries (PPC) was obtained by dividing FCD by TCD.

Intraclass correlation coefficient was performed on a sample dataset to ascertain interrater reproducibility agreement between examiners for TCD and FCD (intraclass correlation coefficient = 0.971; *p* < 0.001). All microcirculation datasets were analyzed by the same investigators (JW, DMJM) and one investigator (JW) was calibrated for ICC by matching analysis from an experienced investigator (DMJM).

### 2.7 Microvascular flow index

The overall microvascular flow index (MFI) score was determined by assessing the predominant flow type in each quadrant as being absent (score 0), intermittent (score 1), sluggish (score 2), or normal (continuous) (score 3) [21,22].

#### 2.7.1 Angioarchitecture

Each isolated image frame was scored and classified according to angioarchitecture classification (AAC) based on either having an appearance of an array of hairpin-like capillary loops (score 1), a combination of hairpin-like capillary loops and vascular network (score 2), or a vascular network without hairpin-like capillary loops (score 3) [23]. Patterns of angioarchitecture can be used to identify (subclinically) epithelial thickness changes and analysis methodology allocation. A score of 1, for example, would allocate analysis to an onscreen vascular count per visual field and a score of 3 would require more complex analysis using specialized algorithms.

#### 2.7.2 Tissue oxygen saturation

InSpectra™ StO_2_ Tissue Oxygen Monitor (ISTOM; Model 650, Hutchinson Technology Inc., Hutchinson, MN, USA) is an FDA-approved bedside method used to measure tissue oxygen saturation (StO_2_) levels. This system operates with near-infrared spectroscopy (NIRS), using a 15-mm optical sensor that emits and detects reflected near-infrared wavelengths (700–1000 nm). The amount of light that is reflected to the sensor following tissue transillumination is dependent upon the oxygen saturation of chromophores, e.g., hemoglobin and myoglobin. The monitor operates noninvasively with a rapid response that estimates continuously the approximate value of StO_2_ by calculating the overall oxy- and deoxyhemoglobin content in the circulating red blood cells. The ISTOM also provides an estimation of the amount of hemoglobin contained in the sampled area, displayed as absolute tissue hemoglobin index (THI) [[24], [25], [26]].

### 2.8 Procedures and data collection

All measurements were performed in the same examination room kept at a mean ambient temperature of 22° ± 2 °C. Following inclusion, medical history was gathered, and subjects underwent an intraoral examination. Basic systemic hemodynamics including HR, SBP, MAP, DBP, StO_2_, and body temperature (°C) were collected once for each participant at baseline on day 1.

Basic systemic hemodynamics were recorded noninvasively in the left upper arm with the subject in a supine, 30° head-up position and body temperature was recorded inside the left ear. Following hemodynamics and temperature registration, subjects were informed about the two CyTs and hands-on demonstrations were set up to assure that subjects got acquainted with the cooling procedures. Upon cooling the participants were asked to seat themselves in a dental chair in an upright or supine, 70° head-up position. In the sessions using IC the subjects were informed to insert an ounce of ice in their mouth and swirl it around constantly to cool, for as much as possible, the whole mouth including inside of lips and all oral mucosae. They were also briefed to swirl the melted ice slurry that was obtained for a few seconds before it was swallowed or spat out prior to loading again with more ICs. Prior to cooling with the ICD, the proper device size was determined before it was self-inserted by the participant and controlled by the investigators to verify a good fit intraorally. Cooling continued for 30 min in two separate sessions with at least 24 h apart (i.e., day 1 and again on day 2). The participants were requested to refrain from eating or drinking for at least 30 min prior to initiating cooling.

At baseline and following 30 min of cooling with each cooling method, the microcirculation was recorded intraorally on 3 non-keratinized regions of interest (ROI), (A) right buccal mucosa (RBM) at the occlusal level of upper 1st molar, (B) right upper lip mucosa (RULM) and (C) right lower lip mucosa (RLLM) at the incisal level of the upper and lower canines, respectively.

To prevent direct contact between the CC imaging probe and patient saliva during intraoral measurements, a disposable cap was used, and the CC handheld unit was draped with a large latex- and powder-free examination glove (Comfort Nitrile Gloves, Fisher Scientific™, Gothenburg, Sweden) with the tip of the middle finger cut off.

The tip of the probe was kept exactly perpendicular to the targeted tissue locations with the lens of the probe in gentle contact placed flat over the epithelium of the mucosa. The same sequential order for RBM, RULM and RLLM was applied for each measurement acquisition. A modified pen grip was used, and the adjacent teeth were used for finger support to ensure stability during microcirculation recordings. After adjusting proper focus and contrast settings were performed during careful probe advancement and retraction maneuvers while in contact with the mucosa to ensure maximizing the presence of capillaries and avoiding pressure-induced artefacts in the field-of-view. All image acquisitions were performed by an experienced researcher (DMJM). A coinvestigator (JW) operated the CC personal computer manually for clip recordings, adjustments of contrast and focus settings and recording of focal depth (Fd). Fd measures the distance between lens focal point of the probe lens and microcirculation and was used as a surrogate for epithelial thickness.

For each measurement, 3 different steady video sequences of adjacent sites of 4 s each were recorded and averaged to represent each anatomic ROI. All measurements were obtained in a standardized way by the same investigator (DMJM) as described above without any preparations of the oral mucosa, other than removing excess saliva or debris from the operating field through absorption using a cotton swab. Following image acquisition all data files was exported and stored on the panel computer and analyzed off-line.

Subsequent measurements of tissue oxygen saturation (StO_2_, THI) were obtained by the same experienced operator (DMJM) using the exact same approach and sequence of measurement acquisition (i.e., RBM, RULM and RLLM) used for the microcirculation measurements. The *trans*-illuminating NIRS light probe was positioned gently on the intraoral mucosa in the specified ROI whereby the highest stable value shown was recorded. Finally at the end of the study following all measurements the participants were asked which of the two cooling methods they preferred.

## 3 Statistical analyses

For comparing oral cooling response in buccal microcirculation (i.e., capillary density), a paired-samples *t*-test with an effect size of 1.2, two-tailed testing with an alpha of 0.05, and a power of 90% results in a required sample size of 10 (calculated using G*Power version 3.1.7) [27]. Normality assumption was controlled using the Shapiro-Wilk test and Gaussian distribution was confirmed for the tested variables. Descriptive data was presented with means±standard deviations (SD). All the parameters related to microcirculation (Fd, TCD, FCD, PPC, MFI, AAC) and tissue oxygen saturation (StO_2_, THI) were analyzed using a paired samples *t-*test. In the assessment of tolerability, i.e., “which of the two cooling methods did you prefer?” a two-sided sign test (McNemar's test) was used. A *p* value < 0.05 was considered statistically significant. All dataset calculations and statistical tests were performed using the IBM® SPSS® Statistics software package (IBM SPSS Statistics version 24, IBM, Armonk, NY).

## 4 Results

**Fig. 2A–F:**
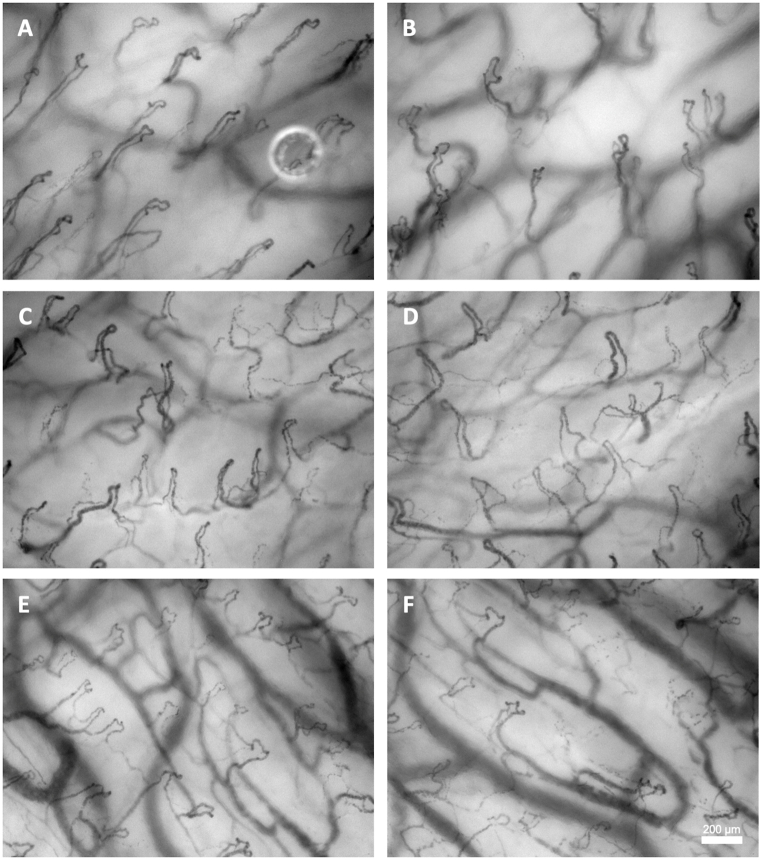
Microcirculatory images obtained with the CytoCam Video Microscope before (left column) and 30 min after (right column) oral cooling (cooling method not specified). (AB) right buccal mucosa at the occlusal level of upper 1st molar; (CD) right upper lip mucosa at the incisal level of the upper canine; (EF) right lower lip mucosa at the incisal level of the lower canine.

A total of 18 subjects were evaluated for study participation, of whom 13 (13/18; 72%) fulfilled the inclusion criteria. Subsequently 3 out of the 13 (3/13; 23%) were excluded due to the use of Swedish snuff. A summary of subject characteristics, basic systemic hemodynamics, body temperature and procedural times is presented in Table 1. All subjects endured 30 min of intraoral cooling with both cooling methods (i.e., IC and ICD). Three video clips per ROI (RBM, RULM, RLLM) were obtained with the CC and NIRS device at baseline and at the 30min follow up with both IC and ICD on day 1 and again on day 2, accounting for a total of 360 oral mucosal microcirculation measurements and 360 oxygen saturation measurements. The oral microcirculation images were of excellent quality and provided good contrast and resolution of all capillaries. Capillaries were clearly visible and could be easily counted (Fig. 2A–F).

**Table 1:**
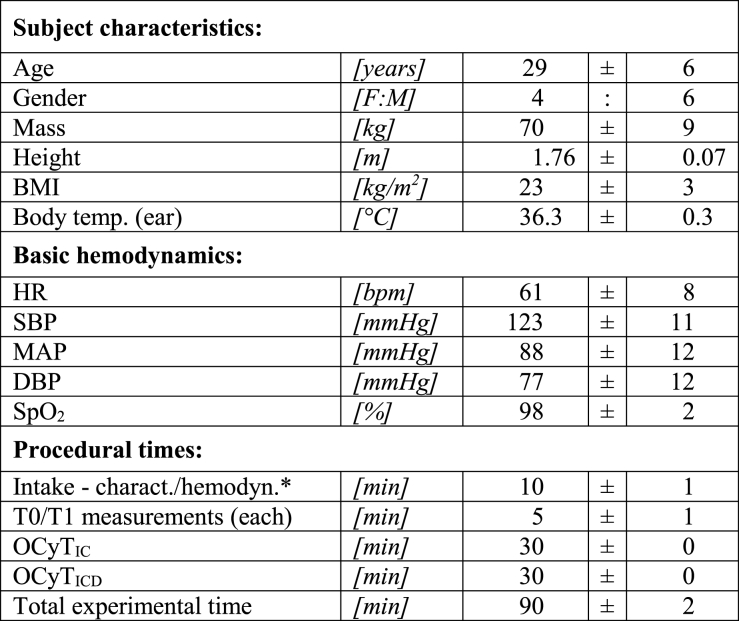
Summary of baseline datasets for subject characteristics; basic hemodynamics; and procedural times. All variables are presented with means ± SD.

BMI body mass index; HR heart rate; bpm beats per minute; SBP systolic blood pressure; MAP mean arterial pressure; DBP diastolic blood pressure; mmHg millimeters of mercury; SpO_2_ peripheral capillary oxygen saturation; T0 baseline time point; T1 follow-up time point; OCyT_IC_ oral cryotherapy with ice chips; OCyT_ICD_ oral cryotherapy with intraoral cooling device; SD standard deviation; *collected once for each participant at baseline.

**Fig. 3:**
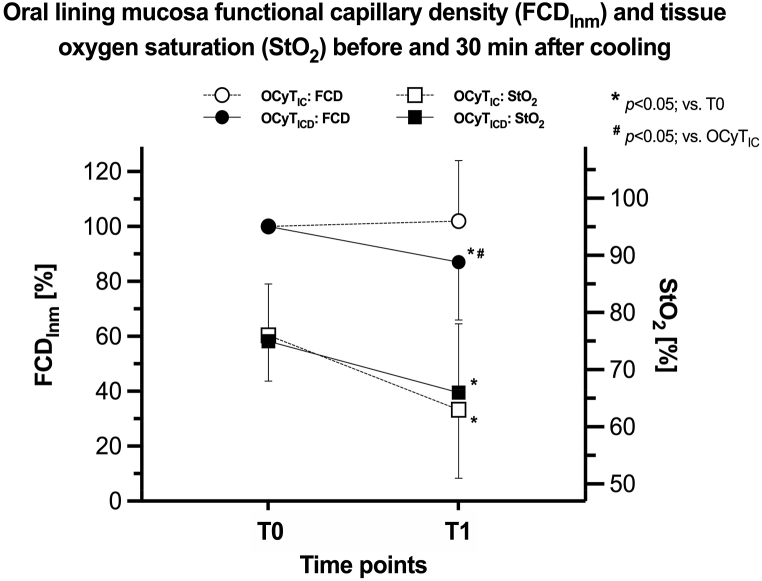
Presents a composite graph illustrating the results of pooling all the oral microcirculatory functional capillary density (FCD) and local tissue oxygen saturation (StO_2_) before and 30 min after cooling with ice chips and intraoral cooling device. T0 baseline time point; T1 follow-up time point; OCyT_IC_ oral cryotherapy with ice chips; OCyT_ICD_ oral cryotherapy with intraoral cooling device. *p < 0.05; vs. T0, ^**#**^*p* < 0.05; vs. OCyT_IC_.

At baseline when data for the 3 ROIs (i.e., RBM, RULM and RLLM) were grouped and mean values were obtained, no statistically significant differences were found between IC and ICD for FCD or StO_2_ (Fig. 3).

In the sessions using IC a mean increase of 2% points (PP) was observed for FCD at follow up time point compared to baseline. However, this increase did not reach statistical significance. The same analysis for ICD revealed a mean decrease of 13 PP (*p* < 0.05). Hence, when FCD was compared between the two cooling methods at the follow up time point, a difference of 15 PP was observed (*p* < 0.05; Fig. 3).

A statistically significant decrease in StO_2_ PP was observed following 30 min of cooling for both IC (13; *p* < 0.05) and the ICD (10; *p* < 0.05) respectively, demonstrating a significant difference for each cooling method versus baseline. The difference of 3 PP between the IC versus ICD was however not statistically significant.

Table 2 summarizes the distribution of all registered microcirculation parameters: Fd, TCD, FCD, PPC, MFI, AAC and the tissue oxygen saturation variables (StO_2_, THI) at baseline and 30 min after cooling with IC and ICD for each ROI. Fd increased significantly in the RBM for IC and ICD, 53 μm (*p* = 0.03) and 74 μm (*p* = 0.01), respectively. In all ROIs, regardless of cooling method, TCD and FCD were equal at baseline and at the follow up. PPC remained 100% and hemodynamic functionality in capillaries remained unchanged throughout the study. In the sessions using IC, FCD increased with 1 cpll/mm^2^ in both the RBM and the RULM whereas it decreased with 1 cpll/mm^2^ in RLLM. However, none of the differences were statistically significant. In contrast, when the corresponding comparison was made for cooling with the ICD, a reduction of 3 cpll/mm^2^, 13% was seen in the RBM (ns); whereas a reduction of 5 cpll/mm^2^ (*p* = 0.012) (20%; *p* = 0.021) and 2 cpll/mm^2^ (*p* = 0.042) (7%; *p* = 0.049) was observed in the RULM and the RLLM, respectively. MFI remained unaltered in all ROIs after cooling with both IC and the ICD, showing continuous blood flow. The same AAC was observed in all microcirculation images, array of capillary loops.

**Table 2:**
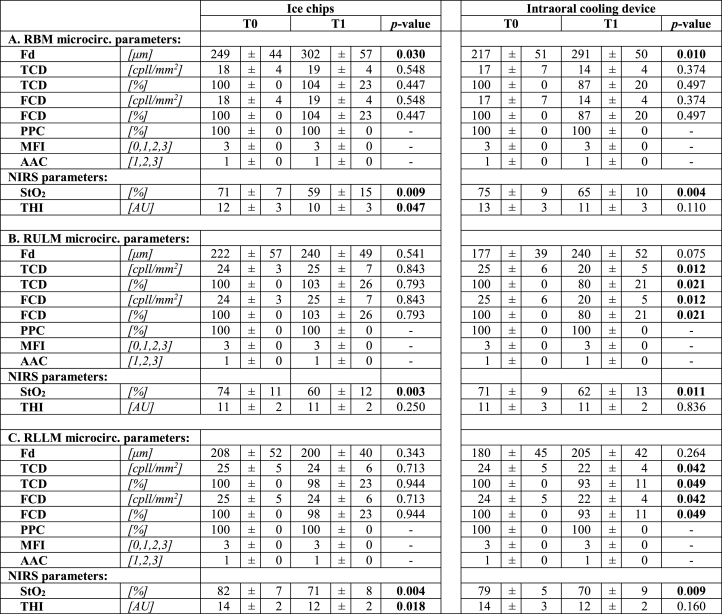
Overview of oral microcirculatory measurement and tissue oxygen saturation (NIRS) datasets from (A) the right buccal mucosa (RBM); (B) right upper lip mucosa (RULM); and (C) the right lower lip mucosa (RLLM) acquired at baseline (T0) and after 30 min (T1) of oral cooling with ice chips and intraoral cooling device. Datasets are presented with mean ± SD. A *p*-value ≤0.05 was considered statistically significant.

AAC angioarchitecture classification; RBM right buccal mucosa; RULM right upper lip mucosa; RLLM right lower lip mucosa; Fd focal depth; TCD total capillary density; FCD functional capillary density; PPC proportion of perfused capillaries; MFI microvascular flow index; NIRS near-infrared spectroscopy; StO2 tissue oxygen saturation; THI tissue hemoglobin index; SD standard deviation.

A statistically significant difference in StO_2_ was found for both IC and ICD following 30 min of cooling. In the cooling sessions using IC, StO_2_ declined in PP by 12 (*p* = 0.009), 14 (*p* = 0.003) and 11 (*p* = 0.004) in the RBM, RULM and RLLM respectively. The corresponding figures for the ICD were 10 PP (*p* = 0.004), 9 PP, (*p* = 0.011) and 9 PP, (*p* = 0.009) respectively for RBM, RULM and RLLM. The estimated THI in the sampled areas showed a statistically significant difference in only the RBM (*p* = 0.047) and RLLM (*p* = 0.018) ROIs when cooling with IC.

Using a McNemar's test, a statistically significant difference was found regarding the participants’ preference. The ICD was preferred over IC by 9 out of 10 participants (90%; *p* = 0.021).

## 5 Discussion

The results of this investigation showed that oral cooling with the ICD was able to significantly reduce tissue perfusion through a reduction in FCD. Interestingly the results also revealed that in both cooling strategies StO_2_ was significantly reduced.

Alterations in microcirculatory parameters have been addressed in several studies adopting the CC. These studies have demonstrated the method's clinical applicability for chairside assessment of the oral mucosal microcirculation [28,29]. The InSpectra™ StO_2_ Tissue Oxygen Monitor system has also demonstrated good clinical applicability for monitoring StO_2_ levels [[24], [25], [26]]. Microcirculation in the oral lining mucosa represented as FCD was found to be unaltered with IC whereas a decline was observed with the ICD. In another previous study in healthy volunteers, the cooling temperatures obtained by the two methods was found to be comparable [17]. Therefore, no difference with regards to the FCD variable was expected between IC and ICD in the present study. The observation that FCD for IC was unaltered at the follow-up time point could be explained by shivering, a biological mechanism which is characterized by increased muscle activity when exposed to cold. Physiologic shivering produces heat to maintain tissue homeostasis. Shivering has been studied in skeletal muscles in relation to skin temperature reduction [30] but this phenomenon is most likely also triggered in the oral mucosa following excessive cold stimuli as when IC is used as a cooling method. Thus, shivering may lead to vasodilation in order to counteract the negative effect of low tissue temperatures [31]. This may be the reason why FCD at after 30 min of cooling for IC was returning to a level comparable with that observed at baseline. To put StO2 reductions into context, shivering requires metabolic energy and the demand for oxygen consumption increases, this may explain the observed decrease in StO_2_ found in both the IC and ICD cooling approaches.

Data concerning the ICD showed that after 30 min of cooling with the ICD, FCD was significantly reduced in 2 out of 3 ROIs. This may be explained by considering that cooling with the ICD at 8 °C is more dispersed over a larger tissue surface area and therefore less likely to trigger shivering to the same extent as intense cold spots perceived by IC. For example, the ICD adheres to a larger surface area in the buccal mucosa as compared to the lips and this can be one such explanatory factor in terms of cold dissipation.

Vasoconstriction remains the generally accepted protective mechanism elicited by OCyT, this was proposed in another study evaluating IC for prevention of chemotherapy-induced OM [5]. However, the different results in FCD between the two CyTs at the follow up time point, suggest that additional mechanisms may instigate the positive effects associated with OCyT. Compared to the results concerning FCD, the decrease in StO_2_ showed a similar pattern for both methods following 30 min of cooling. Thus, no general causal relationship seemed to exist between the microcirculation and tissue oxygen saturation levels. This would presumably have been the case, if StO_2_ reduction occurred solely as a consequence of decreased oral microcirculation. Interestingly, it is possible that cooling of the tissue elicits a temporary state of suspended animation in which metabolism would be temporarily reduced to a point in which oxygen consumption would be diminished resulting in higher measured levels of StO_2_. This was unfortunately not observed in the StO_2_ measurements in the present study. Therefore, it remains to be clarified what caused the decline in StO_2_ and if the decline *per se* leads to sequential mechanisms of relevance for tissue preservation, e.g., lower metabolism in response to reduced tissue oxygen levels. Recently, it was found that cell viability was better preserved when incubated at lower temperatures, assuming that reduced metabolic activity may be a possible explanation for prevention of cytotoxic cell damage [32]. On the contrary, it may also be the case that the decline in StO_2_ occurs because of compensatory increased cellular mechanisms, induced by cooling [33,34]. THI was calculated based on an algorithm of StO_2_ changes. Thus, a similar decrease in THI was anticipated as StO_2_ was reduced following cooling. Such correlation was, however, not observed in the RULM using IC nor in any of the regions using the ICD. More studies are warranted to further confirm results for THI in these regions. The ability to tolerate OCyT is often overlooked in most previous studies concerning OM. ICD was preferred over IC by the majority of the participants in this study. This finding was also similar to another previous study in which a crossover designed study was used to compare the two cooling methods [17].

To the best of our knowledge, the present study is the first to assess oral microcirculation in conjunction with cooling, using techniques that enable real-time visualization. The main advantage of this trial was the crossover design. This, as each crossover subject serves as their own control and thus reduce the influence of cofounding covariates [35]. In addition, crossover designed studies are statistically more efficient and require fewer participants as compared to non-crossover designs. Another advantage was the evaluation and subsequent comparison of two different cooling methods which made it possible to evaluate oral hemodynamics in response to different cooling temperatures. There are also some limitations in this study that need to be acknowledged. First, the number of participants included in this study was small but given the statistical power of 90%, the sample size was sufficient to detect a difference in capillary density between the two cooling methods, i.e., the primary endpoint. Second, this study was carried out in healthy volunteers and although unlikely, both microcirculation and tissue oxygen saturation may be different in patients with cancer.

## 6 Conclusion

Both microcirculation parameters and tissue oxygen saturation are altered in conjunction with oral cooling, indicating their potential mechanistic contribution towards cryoprevention of OM. Mapping the mucosal events that occur during OCyT could be a valuable footing for the development of new treatment strategies for prevention of chemotherapy-induced OM.

## Funding source

This work was supported by BrainCool AB. The funding source was not involved in study design, collection, analysis, interpretation of data, preparation of the manuscript or in the decision to submit the manuscript for publication.

## Author contribution statement

J. Walladbegi and D.M.J. Milstein: Conceived and designed the experiments; Performed the experiments; Analyzed and interpreted the data; Contributed reagents, materials, analysis tools or data; Wrote the paper. J.E. Raber-Durlacher: Contributed reagents, materials, analysis tools or data; Wrote the paper. M. Jontell: Conceived and designed the experiments; Analyzed and interpreted the data; Wrote the paper.

## Data availability statement

Data will be made available on request.

## Declaration of competing interest

The authors declare the following financial interests/personal relationships which may be considered as potential competing interests: Dr. J. Walladbegi reports personal fees from BrainCool AB. Dr. M. Jontell reports consultation fee from Braincool AB and grants from Braincool AB. Drs. J.E. Raber-Durlacher and D.M.J. Milstein have no conflicts of interest to disclose.
